# Burden of Mortality and Disease Attributable to Multiple Air Pollutants in Warsaw, Poland

**DOI:** 10.3390/ijerph14111359

**Published:** 2017-11-08

**Authors:** Piotr Holnicki, Marko Tainio, Andrzej Kałuszko, Zbigniew Nahorski

**Affiliations:** 1 Systems Research Institute, Polish Academy of Sciences, 01-447 Warsaw, Poland; mkt27@medschl.cam.ac.uk (M.T.); kaluszko@ibspan.waw.pl (A.K.); nahorski@ibspan.waw.pl (Z.N.); 2 UKCRC Centre for Diet and Activity Research (CEDAR), MRC Epidemiology Unit, University of Cambridge, Cambridge CB2 1TN, UK; 3 Warsaw School of Information Technology (WIT), 01-447 Warsaw, Poland

**Keywords:** air pollution, exposure, mortality, disability-adjusted life years (DALY), health impact assessment

## Abstract

Air pollution is a significant public health issue all over the world, especially in urban areas where a large number of inhabitants are affected. In this study, we quantify the health burden due to local air pollution for Warsaw, Poland. The health impact of the main air pollutants, PM, NO_X_, SO_2_, CO, C_6_H_6_, BaP and heavy metals is considered. The annual mean concentrations are predicted with the CALPUFF air quality modeling system using the year 2012 emission and meteorological data. The emission field comprises point, mobile and area sources. The exposure to these pollutants was estimated using population data with a spatial resolution of 0.5 × 0.5 km^2^. Changes in mortality and in disability-adjusted life-years (DALYs) were estimated with relative risk functions obtained from literature. It has been predicted that local emissions cause approximately 1600 attributable deaths and 29,000 DALYs per year. About 80% of the health burden was due to exposure to fine particulate matter (PM_2.5_). Mobile and area sources contributed 46% and 52% of total DALYs, respectively. When the inflow from outside was included, the burden nearly doubled to 51,000 DALYs. These results indicate that local decisions can potentially reduce associated negative health effects, but a national-level policy is required for reducing the strong environmental impact of PM emissions.

## 1. Introduction

Ambient air pollution causes one of the biggest environmental health challenges in many Global cities. According to the World Health Organization [1], air quality in the majority of urban agglomerations—especially in low- and middle-income countries—do not meet the respective air quality guidelines [2]. Air pollutants, and fine particulate matter (PM_2.5_), in particular, are emitted into the atmosphere from many sources and cause a multitude of environmental and health effects. Some of the health effects (stroke, heart disease, lung cancer, chronic and acute respiratory diseases, including asthma) are mainly caused by fine fractions of particulate matter [3,4]. A high concentration of this type of air pollution is estimated to cause more than three million premature deaths worldwide each year [1].

As many other European agglomerations, Warsaw also suffers from high concentrations of air pollutants which are typical of the urban environment. These include particulate matter, sulfur dioxide (SO_2_), nitrogen oxides (NO_X_), carbon monoxide (CO), benzo(a)pyrene (BaP), and heavy metals (Pb, As, Cd, Ni), as well as polycyclic aromatic hydrocarbons (PAHs). In practice, the adverse impact of some particular pollutants on urban air quality depends on several individual factors, such as the city location, topography, the structure of the emission field, meteorology, etc. In Warsaw, the composition of the main polluting species, their spatial distribution, and their maximum values also reflect the peculiar structure of the local emission field, which is determined by two dominating factors.

The first factor relates to coal, which is the main fossil fuel used in Poland for power generation and for residential heating [5]. The majority of Warsaw is covered by the district heating system, but in some peripheral districts and the neighboring area coal-fired, small-scale heating installations are used, which considerably contribute to the worsening of air quality. This category of emission sources is responsible for particulate matter pollution (especially PM_2.5_), SO_2_, some heavy metals and BaP. The BaP pollution, which mainly originates from the municipal sector, exceeds the limit value of the annual mean BaP concentration [3] in the whole area of the Warsaw agglomeration.

The second factor relates to the key air pollution category, traffic. For example, in last decade number of cars registered in Warsaw increased by 80% [5]. This trend is different from many other European cities, but representative for many global cities in low- and middle-income countries. Traffic-originated emission is mainly responsible for NO_X_, CO, benzene (C_6_H_6_) and partly for Pb concentrations, but it also contributes to particulate matter PM_10_ pollutions, mainly via the re-suspended particles [6,7]. In particular, basing on the reports [5,8], related to the years 2005 and 2012, respectively, concentrations of NO_X_ and PM_10_ have been on the rise during the last decade, and both exceed the annual average concentration limits.

The external inflow of some pollutants originating from distant sources also contributes significantly to the resulting air pollution in Warsaw, which mainly relates to the fine fractions of particulate matter, as shown in [9,10,11].

This study quantifies health burden caused by air pollution in Warsaw. We first estimate the population average exposure for multiple air pollutants (particulate matter (PM), NO_X_, SO_2_, CO, C_6_H_6_, BaP and heavy metals (Pb, As, Cd, Ni)), and then predict changes in attributable deaths and disability-adjusted life-years (DALYs) due to this exposure. The modeling results of air pollution, which are utilized in an analysis of the negative health effects, are presented in more details in [5] and are not repeated in this study. Here we only recall the main assumptions and findings relevant for predicting health burden.

## 2. Methods

### 2.1. The Study Area and Spatial Resolution

The base of this study is an air quality analysis for the Warsaw agglomeration from the year 2012, presented in [5,12]. To simulate pollution dispersion processes, the Gaussian puff model CALPUFF v.5, [13], frequently applied in regional/urban scale analysis of air quality [12,14] and recommended by US EPA [15], was used. It is a multilayer, non-stationary model designed for calculating concentrations of many substances, emitted by different types of sources. Meteorological fields were re-analyzed by the Weather Research and Forecasting Model (WRF) model, and the National Center for Atmospheric Research [16], and assimilated to the final resolution grid by the CALMET meteorological, cooperating preprocessor. The aim of the simulation was to obtain the spatial maps of the year average concentrations of the main urban pollutants (see Table S1 in Supplementary Materials), to show districts/areas where the pollution limits were exceeded and to identify emission sources responsible for these violations. See [5,8] for uncertainty estimates and assessment of the model’s performance.

### 2.2. The Structure of the Emission Field

**Figure 1:**
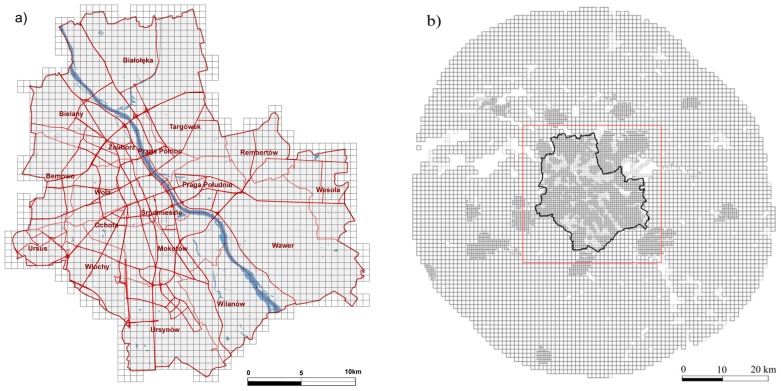
The study domain: (**a**) resolution of the receptor area; (**b**) resolution of the total emission area.

The Warsaw Metropolitan Area—about 520 km^2^ within the administrative borders and with a total population of 1,715,517 inhabitants [17] in the year 2012—is shown below in Figure 1 (for detailed population structure see Supplementary Materials Table S2). The study area was discretized for the numerical analysis with a homogeneous grid of 0.5 × 0.5 km^2^. To take into account specific types of the different emitters, the total emission field was split into the following categories of sources: point (high and low emission height separately), area, and line (mobile). A separate class of the high point sources was comprised of the power/heating plants, which operate within the district heating system and are used in the main part of the agglomeration. Thus, the aggregate emission field consisted of the categories listed here, with the respective quantity of the individual sources in each category:

- High point sources (24)—energy generation;
- Low point sources (3880)—industrial plants;
- Area sources (6962)—residential combustion;
- Line sources (7285)—urban road traffic;
- Boundary conditions (the inflow of some pollutants due to the regional/national level emission based on the results from the European scale EMEP model [3]).

The total emission field encompassed the Warsaw area within its administrative borders and the surrounding belt of approximately 30 km wide (see Figure 1b). The locations of the point sources were identified by their geographical coordinates. The area and line sources were represented as basic grid emission squares, 0.5 × 0.5 km^2^, inside the Warsaw administrative borders (Figure 1a), and also in the aggregated grid, 1 × 1 km^2^, of the surroundings (Figure 1b). The local city areas in the suburban region were also represented by the nested fine resolution grid, as shown in Figure 1b. 

The computed annual mean concentrations of the polluting compounds listed in (Supplementary Materials Table S1) have been recorded at 2248 receptors points, which coincided with the central points of the spatial resolution elements shown in Figure 1a.

### 2.3. Concentrations and Population Weighted Exposure of Air Pollutants

**Figure 2:**
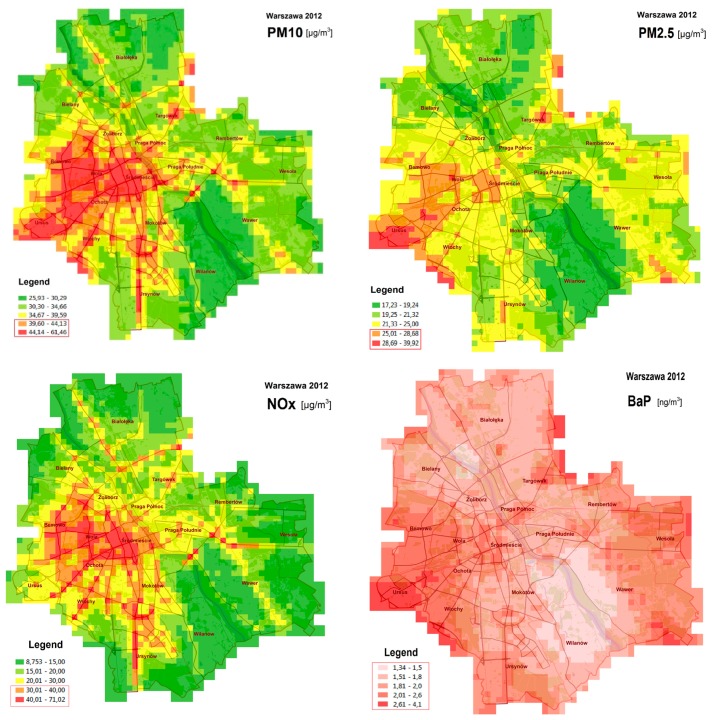
The annual mean concentrations maps for the year 2012 (cf [5]), where the limit values are exceeded.

The annual mean concentrations of NO_X_, PM_10_, PM_2.5_ and BaP exceeded the European Union limit values [2,18] in some districts [5]. The respective concentration maps are shown in Figure 2. The other compounds listed in Supplementary Materials Table S1, did not violate the air quality standards [5], but they also contributed to the final adverse health effects.

**Figure 3:**
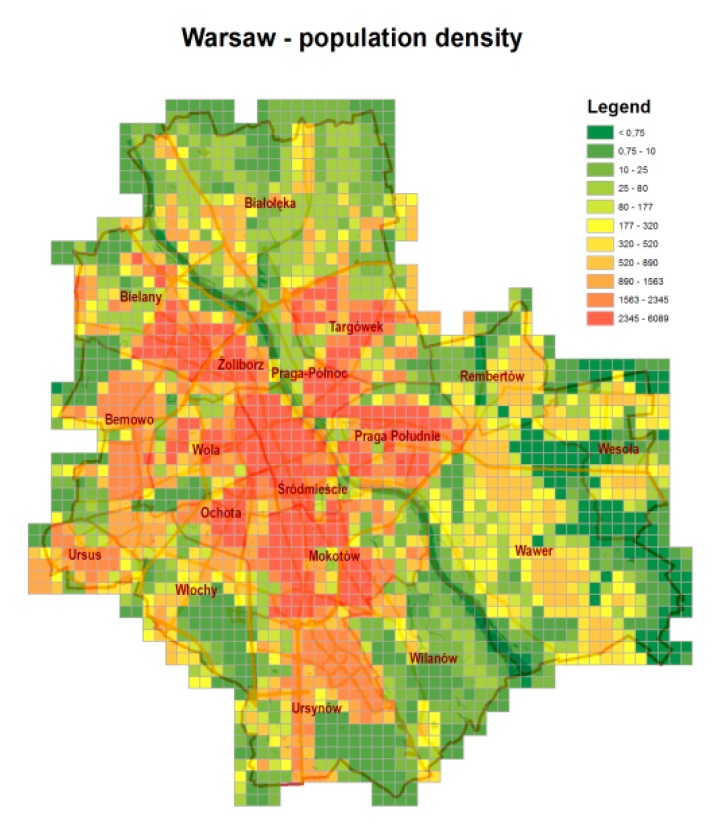
Population density map for Warsaw (as compiled in [19]).

Quantification of these effects was based on the population average concentration (exposure) of the pollutants considered. Figure 3 presents the population density map of Warsaw [19]. The map for the year 2005 was previously used in [20], and was modified for the year 2012 according to [17,21] data. The spatial resolution applied in the population density map is the same as that used in the forecasting model computations (0.5 × 0.5 km^2^). The legend on the map represents the number of inhabitants in one elementary resolution square.

The population average exposure (*E*) for the individual emission sources and the specified pollutant, was calculated with the following formula:

(1) $$E_{k,j}=\frac{1}{Pop}\sum_{i}C_{i,k,j}\cdot Pop_{i}$$

where $E_{k,j}$ is exposure, $C_{i,k,j}$ is concentration, $Pop_{i}$ is the receptor population, *i* is the receptor index, *j* is the pollutant index, and *k* is the emission source index within emission category. The aggregated exposure index for each pollutant within the emission category was obtained by summing up in (1) with respect to the emission sources k.

(2) $$E_{j}=\frac{1}{Pop}\sum_{k}\sum_{i}C_{i,k,j}\cdot Pop_{i}$$

The distribution of the population density in (1) and (2) plays the role of the weight function, hence, the unit of exposure in both formulas is the same as for concentration, (µg/m^3^).

### 2.4. Estimation of Health Burden

The estimation of health burden followed similar methods as described in [20,22]. For gaseous air pollutants and metals, the health risks were estimated for an individual pollutant. For the particulate matter, the health risks were calculated separately for two size fractions: PM_2.5_ and PM_2.5–10_. Thus, we assumed that the toxicity of the particles varied between primary (PM_2.5_) and coarse (PM_2.5–10_) fractions of the PM, but not between the source or chemical composition. The details on burden of disease calculations, exposure-response functions, equations and data sources are all described in Supplementary Materials Tables S3 and S4 [23,24,25,26,27,28,29,30,31,32,33,34,35,36,37,38,39,40,41,42,43].

Two measures of health were used: the number of attributable deaths, and disability-adjusted life-years (DALY). The advantage of the DALY measure is that it combines mortality and morbidity impacts into one measure of health, allowing for comparison between, for example, mild mental retardation caused by Pb, and increased mortality caused by PM_2.5_.

For the background DALY and mortality data, we used the year 2013 Global Burden of Disease [44] country file for Poland (Supplementary Materials Table S5). The burden data was estimated from national data by assuming that age and gender specific death and DALY rates were the same in Poland and in Warsaw (see population data in Supplementary Materials Table S2). The health calculations were done with the Monte Carlo simulation program Analytica, version 4.6. (Lumina Decision Systems, Inc., Los Gatos, CA, USA). Uncertainty was propagated through the model with 50,000 iterations.

## 3. Results

### 3.1. Concentration and Exposure

The spatial distribution of exposure values for selected pollutants, representing the line and area emission categories in the considered domain is shown in Supplementary Materials Figure S1. All the sources presented were split into two groups: those located inside the square domain indicated in Figure 1b (blue dots) and those located outside this square (red dots). The aim was to assess the share of emission sources located outside the close vicinity of Warsaw. For the line emissions (left panels), the dominating share of the intra-urban sources could be seen, including high-traffic roads in the close vicinity of Warsaw. For the area emissions, the share of the intra-urban sources was low, mainly due to residential emissions of the peripheral districts (right panels). On the other hand, a significant contribution of the sources located in the direct vicinity of Warsaw (blue color) could be observed. This was not only due to their emission intensity, but also to results from a coarse spatial resolution in this case, where each emission source was represented by an element of 1 × 1 km^2^, instead of 0.5 × 0.5 km^2^, as for other sources.

The trans-boundary inflow contributed significantly to the final exposure. Aerosols ${\mathrm{SO}}_{4}^{2-}$ and NO${}_{3}^{-}$ were the secondary pollutants (Supplementary Materials Table S1), where the share of the local sources was minor, mainly due to the time which is required for aerosol formation. The contribution of the aerosol’s inflow from distant sources was greater due to the longer periods of time spent in the atmosphere, where they were transported and transformed. Also, the contribution of the inflow particulate matter, which contained aerosols as components, was considerable. 

The population weighted concentration (exposure) for the studied air pollutants is presented in Table 1 for four local emission categories and the trans-boundary inflow from distant emission sources. For most pollutants, local emission sources caused larger exposure than external inflow from outside the study area. The main exceptions were secondary sulfate and nitrate aerosols for which inflow contribution was dominate in the resulting exposure (76% for ${\mathrm{SO}}_{4}^{2-}$ and 81% for NO${}_{3}^{-}$). Due to the spatially limited receptor area, the time interval during which the local pollutants remained in the domain was too short for complete transformation. The time required for aerosol formation is a key factor in this case. In the cases of PM_2.5_ and CO, almost half of the exposure was also due to external inflow.

### 3.2. Health Burden

Air pollution was estimated to cause approximately 2800 (95% CI: (Confidence Interval) 2100 to 3500) attributable deaths per year in the study area (Table 2) and 51,000 (95% CI: 39,000 to 62,000) DALYs (Table 3). Approximately 82% of the total attributable deaths were due to PM_2.5_ air pollution, and 16% due to NO_X_. About 1% of the deaths were due to all other pollutants. Air pollution influx from outside of the study area caused 45% of the deaths, and local emissions caused 55% (Table 2). Of local emission sources, the area sources (residential) were the most important, followed by the line sources (traffic) (Table 2). Point sources (high and low combined) caused about 1% of the attributable deaths. The DALY results presented in Table 3 were similar to attributable deaths, with most of the DALYs (84%) being due to PM air pollution, followed by NO_X_ (14%). From the morbidity outcomes, chronic bronchitis (COPD) caused the highest health burden.

## 4. Discussions

In this study, we quantified the health burden due to air pollution in the city of Warsaw, Poland, with the high resolution of 0.5 × 0.5 km^2^. The emission field comprised the city territory and the area surrounding the city with the diameter of 90 km, as well as the pollution inflowing from outside of the study area. Several pollutants typical of the town atmosphere were considered, including particular matters, oxides, and heavy metals, as well as benzene and benzo(a)pyrene. Due to differentiated land use characteristics of the town, and different types of urban development, the pollution was very diverse in different town quarters, thus the high resolution estimation improved the quality of results. To quantify the health effect, the health risk was calculated for each individual pollutant.

### 4.1. Meaning of the Study

This study provides important background information for developing mitigation strategies for air pollution in Warsaw. The magnitude of the health burden, 2800 deaths per year and 51,000 DALYs per year, indicate that air pollution is a significant environmental health problem in Warsaw. These represent 15% and 9% of all deaths and DALYs, respectively, in the study area (Supplementary Materials Table S5). Approximately 45% of the attributable deaths were due to air pollution inflow from outside Warsaw, and 55% were due to local emissions sources, indicating that local, national and international mitigation strategies are required in order to reduce the health burden. From local sources, the area sources, representing residential emissions, caused 47% of the burden and the linear sources (traffic) caused 50%. This clearly indicates that local mitigation actions should target these two emission categories, while point sources had a nearly insignificant direct impact on the health burden.

The study also provides information on the relative weight of different air pollutants for causing health risks, and information regarding the health outcomes they cause. For both attributable deaths and DALYs, PM_2.5_ caused almost the entire health burden, and for DALYs, most of the health burden due to PM_2.5_ was associated with non-accidental mortality. Thus, most important air pollution is PM_2.5_, which causes non-accidental mortality. Other pollutants and health outcomes had minor impacts on health. This result is similar to the European Environmental Agency (EEA 2015) Air Quality in Europe report that estimated attributable deaths due to PM_2.5_, ozone (O_3_) and NO_2_. For Poland, the number of deaths were 44,600, 1100 and 1600, respectively, for the three different pollutants. Also, in their analysis most of the attributable deaths (94%) were due to PM_2.5_. Similarly, the European Environmental Burden of disease study that included PM_2.5_, benzene, and lead, together with several other environmental stressors, concluded that most of the health burden was due to PM_2.5_.

Although the presented results relate specifically to the Warsaw agglomeration, the conclusions are likely applicable to other central-eastern European cities, and also to many cities in low- and middle-income countries around the world. Warsaw has seen rapid the growth of private car ownership in the past decade, while at the same time houses are still warmed by coal [5]. In addition to local emissions, the influx of pollutants from outside the city also plays an important role in reducing the air quality.

### 4.2. Strengths and Limitations of the Study

The strength of this study is the use of a well-established dispersion modeling system based on CALPUFF and emission data, the combination of fine scape population data with the resulting air pollution concentration, and estimating the health burden for multiple air pollutants and health outcomes. The main strength lies in a combination of these methods to product one assessment with one purpose.

The main limitation of the study is the lack of ozone concentration impact on the considered health burden. Together with PM_2.5_ and NO_2_, ozone is among most important air pollutants from a health effect point of view. The CALPUFF system, due to its linear structure, is not an appropriate modeling tool to analyze tropospheric ozone formation. On the other hand, the acquaintance of ozone concentrations is an important driving force in other urban atmospheric processes. Hence, in this study, the ozone concentrations are based on the measurements [45] for the year 2012. A sequence of hourly observed values at eight stations located in the study area (Figure 1) were entered and interpolated the computational grid. The hourly variability range of the measured ozone concentrations was 3–90 µg/m^3^. The annual mean values were 24–30 µg/m^3^ depending on the measurement point, and the similar mean for the summer period with the highest occurrence of ozone were within the range of 28–38 µg/m^3^. The Polish reference value [45] of hourly ozone concentration is set to 150 µg/m^3^. As indicated in an earlier study [46], ozone is estimated to cause 1100 attributable deaths in Poland, and likely tens or hundreds of cases in Warsaw. Even if we assume that ozone would cause hundreds of attributable deaths in Warsaw, the total health burden in Table 2 would be in the same magnitude. Hence, the health burden due to ozone would be much smaller than that for PM_2.5_, and at maximum in same level with NO_X_. Moreover, lacking the modelling results, it is not known how much of the ozone concentration was due to distant sources, so the contribution of local sources to local level ozone could be even lower.

The attributable deaths and DALYs due to air pollution were calculated by combining the impact of individual pollutants together, although some of the pollutant categories used in this study overlapped. For example, metals disperse through the air in particulate format and the metal emissions were therefore also included in PM emissions. This may have led to overestimation of the impact. However, since the total burden caused by all the heavy metals and BaP combined was still less than 1% of the total burden, it is assumed that the potential impact of double counting is small. In some environments, where the concentrations of heavy metals are higher, the method used here could potentially lead to higher overestimation of the burden.

We also acknowledge that evidence on causality vary between pollutants, being strong for PM_2.5_ and Pb but less so for SO_2_ and NO_X_. Here we assumed that all pollutants considered are causally linked to associated health outcomes, and this could result in overestimation of the true burden if future research would prove otherwise. However, as with the potential double counting, the total burden would likely be estimated to be similar because most of it is due to PM_2.5_, which does have strong epidemiological evidence to back-up causality.

We also estimated the health burden by assuming that the whole population was exposed to the same population average concentration. Due to non-linearity in the exposure-response functions, this will have created a small error in the calculation in comparison to a situation where we would estimate the health burden of each 0.5 × 0.5 km grid separately.

### 4.3. Comparison to Other Studies, Discussing Important Differences in Results

Two previous studies have estimated the health burden due to transport-related air pollution in the same study area with substantially different methods and results. Tainio [22] estimated that transport-related air pollution causes 25,000 DALYs a year (in this study, 15,000 DALYs, Table 3) using methods and data similar to this study. The main reason for the lower burden in this study is the update of the concentration–response function for all-cause mortality for PM_2.5_, [23,30]. Adamkiewicz et al. [47] used roadside measurements to estimate the contribution of local traffic to atmospheric PM_10_ and NO_X_ concentration in the study area, and the Life Cycle Impact Assessment tool ReCiPe (http://www.lcia-recipe.net/) to estimate the health burden due to these two pollutants. Their estimate for health burden is 1700 DALYs, about one magnitude smaller than our estimate. However, if we compare [47]’s results with the health burden caused by PM_2.5–10_ and NO_X_, then the difference in results is much smaller (5400 DALYs in this study versus 1700 DALYs in [47]). This might indicate that [47]’s results are smaller because they didn’t include fine particulate matter (PM_2.5_) in their analysis. 

Few studies have estimated the burden of disease due to air pollution in Poland. In the [19] air quality report, the total attributable deaths in Poland was assumed to be 47,300 deaths per year, and another study estimating impact reported 39,800 attributable deaths in Poland for the year 2000 [46]. When scaled from the population of Poland (38.6 million) to the population of Warsaw (1.72 million), the attributable deaths from each study would be 2100 and 1800 cases per year, respectively, by assuming that the burden was equally distributed around the country. In the Global Burden of Disease Study 2013 [44] the impact of air pollution in Poland was 433,000 DALYs, and similarly, the contribution of Warsaw would have been 19,300 DALYs (versus the 51,000 DALYs estimated in this study). The result from the EEA is similar to this study (2100 versus 2800 deaths) when taking into account that urban areas are more polluted than country areas, on average. The estimate from study [44] is much smaller. The GBD (Global Burden of Disease) Integrated Risk Function (IRF) method [48] used uncertain threshold values between 5.8 and 8.8 µg/m^3^ to set up counter-factual scenarios for five individual disease outcomes (ischemic heart disease (IHD), stroke, chronic obstructive pulmonary disease (COPD), lung cancer, and acute lower respiratory infection). The use of individual diseases might have led to smaller impacts than the use of all-cause mortality, defined as natural mortality in this study. However, without detailed analysis of GBD results, that cannot be quantified.

The results between different pollutants and health outcomes were similar in study [22], which estimated health effects of local transport-related air pollution in Warsaw. Also in that study, most of the air pollution-related health effects were due to non-accidental mortality due to PM_2.5_. However, in the present study the relative contribution of PM_2.5_ was smaller, due to an updated concentration-response function for PM_2.5_. This increased the importance of NO_X_, but had a minor impact on other pollutants. Another study from Finland [49] estimated the burden of air pollution for 14 different pollutants, including all the main pollutants from this study plus ozone. They found out that from the total health burden (33,000 DALYs), 64% was due to PM_2.5_, and 28% due to PM_10_, ozone and NO_2_. Thus, in their analysis, the total contribution of PM_2.5_ alone was slightly smaller than in our study (71%) but within a similar magnitude when taking into account that they included the effect of ozone.

The estimates of Mild Mental Retardation (MMR) presented in Table S6 of Supplementary Materials are based on [39,50]. Relative risk values for cardiovascular disease shown in Table S7 in Supplementary Materials are adopted from [39,41].

### 4.4. Unanswered Questions and Future Research

Only exposure to outdoor air pollution was considered in the assumed home addresses. Most of this exposure to outdoor air pollution occurs indoors and is impacted by the indoor sources of air pollutants. For example, [51] estimated that in Poland 66% of the burden of disease from residential indoor exposure is due to PM_2.5_ from the outside air. Other significant sources were indoor generated PM_2.5_, radon and home dampness.

## 5. Conclusions

The modelling results indicate that air pollution causes 2800 deaths a year in Warsaw. From this 45% are due to inflow from outside the study area, and the rest due to local emissions. From all the local emissions, area sources (residential) caused 46% of the burden and linear sources (transport) caused 52%. The impact of point sources was around 1%. Nearly all the deaths (91%) were due to PM_2.5_, highlighting importance of this pollutant for population health. When morbidity effects were included in the calculations, non-accidental mortality due to PM_2.5_ accounted for about 71% of the total DALYs (36,000 DALYs out of a total impact of 51,000 DALYs).

A large fraction of the PM_2.5_ pollution in Warsaw comes from sources located outside of the Warsaw borders. In this study, about half of the related health risks in Warsaw were due to the local emission sources and the other half due to inflow. Thus, the dominating risk factor relates to high exposure to fine particular matter, coming both from local and external sources. Since Poland is one of a few European Union countries responsible for the highest PM_2.5_ emissions (including BaP) (EEA 2012; 2015), appropriate government decisions are essential for decreasing the health risk level. With reference to the housing sector, policies that could reduce emissions would include, for example: (i) assisted replacement of the coal-fired installations by natural gas ones; (ii) subsidized installations of low-emission coal furnaces; (iii) considerable increase in the coal quality for domestic use. Moreover, since the other emission categories also significantly contribute to the inflow of pollutants (85% of the energy in Poland is generated by coal combustion), an increase in the share of renewable sources on the national scale could also improve air quality in Warsaw, and elsewhere in Poland.

## Figures and Tables

**Table 1:** Population weighted exposure (µg/m^3^) for emission categories.

Pollution	Unit	Point Sources		Line	Area	Local Sources	External Inflow	Total. Exposure
		High	Low					
**SO_2_**	(µg/m^3^)	0.71	0.27	1.32	3.68	5.99	1.46	7.45
**SO_4_**		0.01	0.00	0.05	0.13	0.20	0.64	0.84
**NO_X_**		0.43	0.41	16.10	2.31	19.25	1.86	21.10
**NO_3_**		0.01	0.01	0.53	0.12	0.67	2.87	3.54
**PPM10**		0.06	0.23	1.93	10.30	12.52	10.15	22.67
**PPM10_r**		-	-	9.14	-	9.14	-	9.14
**PPM_2.5_**		0.02	0.10	1.30	8.02	9.44	7.35	16.84
**PPM_2.5__r**		-	-	1.30	-	1.30	-	1.30
**PM10**		0.08	0.24	11.66	10.55	22.52	13.66	36.18
**PM_25_**		0.04	0.12	3.18	8.27	11.60	10.86	22.51
**CO**		0.14	0.48	145.40	7.57	153.60	132.88	281.67
**C_6_H_6_**		0.29	0.12	0.70	0.00	1.11	0.00	1.11
**Pb**		0.00	0.00	0.01	0.01	0.014	0.00	0.015
**As**	(ng/m^3^)	0.00	0.00	0.00	0.73	0.735	0.00	0.735
**Cd**		0.00	0.05	0.01	1.06	1.120	0.04	1.164
**Ni**		0.06	0.11	0.67	3.35	4.194	0.00	4.194
**BaP**		0.01	0.02	0.14	1.12	1.286	0.66	1.946

**Table 2:** Attributable deaths (number of deaths per year) in the study population by source, pollutant and cause of mortality: mean and (95% CI). (Confidence Interval)

Pollutant	Point High	Point Low	Line	Area	Inflow	Total	% of Total Burden
PM_2.5_: Non-accidental mortality	4 (3 to 5)	12 (9 to 14)	388 (291 to 480)	691 (517 to 856)	1210 (906 to 1498)	2304 (1725 to 2853)	82
NO_X_: Non-accidental mortality	7 (2 to 12)	8 (2 to 14)	380 (87 to 665)	35 (8 to 61)	27 (6 to 47)	457 (104 to 800)	16
SO_2_: Lung cancer	1 (−4 to 5)	0 (−1 to 2)	2 (−9 to 12)	3 (−17 to 22)	1 (−7 to 10)	8 (−38 to 50)	0
BaP: Lung cancer	<1	<1	<1	2 (1 to 2)	1 (0 to 1)	3 (1 to 4)	0
Cd: Cancer	<1	<1	<1	1 (0 to 2)	<1	1 (0 to 2)	0
Ni: Cancer	<1	<1	<1	<1	to	<1	0
Pb: Cardiovascular diseases	<1	<1	7 (3 to 12)	4 (2 to 7)	0 (0 to 1)	11 (6 to 21)	0
As: Lung Cancer	<1	<1	to	<1	to	<1	0
CO: Ischemic heart disease	<1	<1	7 (3 to 11)	<1	4 (2 to 6)	11 (4 to 17)	0
C_6_H_6_: Leukemia	<1	<1	<1	<1	to	<1	0
**Total**	**12 (4 to 19)**	**20 (13 to 27)**	**783 (476 to 1085)**	**736 (559 to 903)**	**1244 (938 to 1533)**	**2794 (2111 to 3455)**	**100**

**Table 3:** DALY due to air pollution in Warsaw, by source, pollutant and cause of morbidity or mortality: mean and (95% CI).

Pollutant	P. High	P. Low	Line	Area	Inflow	Total
PM_2.5_: Non-accidental mortality	59 (44 to 73)	182 (136 to 226)	6094 (4563 to 7546)	10,852 (8126 to 13,438)	19,000 (14,228 to 23,528)	36,186 (27,098 to 44,811)
PM_2.5_: Chronic bronchitis (COPD)	5 (1 to 9)	15 (3 to 28)	509 (101 to 927)	906 (179 to 1651)	1586 (314 to 2891)	3021 (598 to 5505)
PM_2.5_: Restric-ted activity days (RAD)	1 (1 to 1)	4 (3 to 4)	122 (110 to 133)	217 (196 to 237)	379 (343 to 415)	722 (654 to 791)
PM_2.5_: LRS symptoms days (School children)	<1	1 (1 to 1)	31 (19 to 43)	55 (33 to 76)	96 (58 to 133)	182 (111 to 252)
PM_2.5_: LRS symptoms days (adult)	1 (0 to 1)	2 (1 to 4)	71 (21 to 134)	126 (37 to 239)	220 (65 to 418)	419 (124 to 797)
PM_2.5–10_: LRS symptoms days (School children)	<1	1 (1 to 1)	88 (54 to 122)	15 (9 to 21)	19 (11 to 26)	123 (75 to 170)
PM_2.5–10_: LRS symptoms days (adult)	1 (0 to 1)	2 (1 to 4)	202 (60 to 384)	36 (11 to 68)	43 (13 to 81)	283 (84 to 538)
PM_2.5–10_: Chronic bronchitis (COPD)	4 (1 to 8)	16 (3 to 29)	1455 (288 to 2652)	256 (51 to 467)	307 (61 to 559)	2038 (403 to 3715)
NOx: Non-accidental mortality	111 (25 to 194)	123 (28 to 215)	5966 (1364 to 10,448)	548 (125 to 959)	423 (97 to 741)	7170 (1639 to 12,557)
SO_2_: Lung cancer	16 (−80 to 105)	6 (−31 to 41)	39 (−197 to 260)	72 (−359 to 473)	31 (−156 to 206)	164 (−823 to 1086)
BaP: Lung cancer	0 (0 to 1)	1 (0 to 1)	7 (2 to 10)	34 (12 to 48)	22 (8 to 31)	63 (22 to 91)
Cd: Cancer	<1	1 (0 to 2)	<1	16 (1 to 40)	1 (0 to 2)	18 (1 to 44)
Ni: Cancer	<1	<1	<1	0 (0 to 1)	to	1 (0 to 1)
Pb: Mild mental retardation (children)	<1	-	8 (3 to 17)	5 (2 to 10)	1 (0 to 1)	13 (5 to 28)
Pb: Cardiovascular diseases (adult)	0 (0 to 1)	-	166 (81 to 301)	100 (49 to 181)	11 (6 to 20)	279 (137 to 507)
As: Lung Cancer	<1	<1	To	<1	to	<1
CO: Ischemic heart disease	<1	<1	98 (38 to 158)	3 (1 to 5)	59 (23 to 95)	160 (62 to 259)
C_6_H_6_: Leukemia	1 (0 to 1)	0 (0 to 1)	3 (2 to 4)	3 (2 to 4)	to	7 (4 to 9)
**Total**	**200 (68 to 326)**	**357 (241 to 469)**	**14,856 (9708 to 19,902)**	**13,241 (10,288 to 16,065)**	**22,196 (17,177 to 26,998)**	**50,849 (39,270 to 62,083)**
**% of total burden due to emiss. source**	0	1	29	26	44	100

## Supplementary Materials

The following are available online at www.mdpi.com/1660-4601/14/11/1359/s1. Table S1: Primary and secondary pollutants considered, Table S2: Population of Warsaw and Poland by age and sex, Table S3: Disability weights and duration data, Table S4: Summary of concentration–response functions used in the study, Table S5: Background burden in the study area, Table S6: Calculation of Mild Mental Retardation (MMR) due to Pb, Table S7: Relative risk values for cardiovascular disease for different blood level Pb’s, Figure S1: Exposure of the selected pollutants attributed to the individual emission sources.

## References

[1] World Health Organization (WHO) Air Pollution Levels Rising in Many of the World’s Poorest Cities. http://www.who.int/mediacentre/news/releases/2016/air-pollution-rising/en/.

[2] CAFE Directive 2008/50/EC of the European Parliament and of the Council of 21 May 2008 on Ambient Air Quality and Cleaner Air for Europe. http://ec.europa.eu/environment/legal/law/5/e_learning/library_documents.htm.

[3] European Environment Agency (EEA) (2012). Air Quality in Europe—2012 Report.

[4] European Environment Agency (EEA) (2015). Air Quality in Europe—2015 Report.

[5] Holnicki P., Kałuszko A., Nahorski Z., Stankiewicz K., Trapp W. (2017). Air quality modeling for Warsaw agglomeration. Arch. Environ. Prot..

[6] Dimitriou K., Kassomenos P. (2014). A study on the reconstitution of daily PM_10_ and PM_2.5_ levels in Paris. Atmos. Environ..

[7] Kiesewetter G., Borken-Kleefeld J., Schöpp W., Heyes C., Thunis P., Bessagnet B., Terrenoire E., Amann M. (2015). Modelling street level PM_10_ concentrations across Europe: Source apportionment and possible futures. Atmos. Chem. Phys..

[8] Holnicki P., Nahorski Z. (2015). Emission Data Uncertainty in Urban Air Quality Modeling—Case Study. Environ. Model. Assess..

[9] ETC/ACM How to Start with PM Modelling for Air Quality Assessment and Planning Relevant to AQD. http://acm.eionet.europa.eu/reports/ETCACM_TP_2013_11_FAIRMODE_guide_modelling_PM.

[10] Wang T., Jerrett M., Sinsheimer P., Zhu Y. (2016). Estimating PM_2.5_-associated mortality increase in California due to the Volkswagen emission control defeat device. Atmos. Environ..

[11] Levy J.I. (2016). Fine Particulate Matter Assessment and Risk Management. Risk Anal..

[12] Holnicki P., Kałuszko A., Trapp W. (2016). The urban scale application and validation of the CALPUFF model. Atmos. Pollut. Res..

[13] Scire J.S., Strimaitis D.G., Yamartino R.J. (2000). A User’s Guide for the CALPUFF Dispersion Model.

[14] Tartakovsky D., Stern E., Broday D.M. (2016). Comparison of dry deposition estimates of AERMOD and CALPUFF from area sources in flat terrain. Atmos. Environ..

[15] United States Environmental Protection Agency (US EPA) (2010). Air Quality Document Technical Support Document: NJ 126.

[16] NCAR (2008). A Description of the Advanced Research WRF Version 3.

[17] GUS Powierzchnia i ludność w przekroju terytorialnym w 2014 r. Główny Urząd Statystyczny.

[18] Ministry of the Environment (ME) (2012). Decree 1031, 24 Aug. 2012, On the Admissible Levels of Some Substances in the Air.

[19] Holnicki P., Tainio M., Kałuszko A., Nahorski Z. (2017). Burden of Disease Due to Air Pollutants Emitted from Urban Sources in Warsaw, Poland.

[20] Tainio M., Holnicki P., Loh M.M., Nahorski Z. (2014). Intake Fraction Variability between Air Pollution Emission Sources inside an Urban Area. Risk Anal..

[21] European Environment Agency (EEA). http://www.eea.europa.eu/data-and-maps/data-population-density-disaggregated-with-corine-land-cover-2000-2.

[22] Tainio M. (2015). Burden of disease caused by local transport in Warsaw, Poland. J. Transp. Health.

[23] Héroux M.E., Anderson H.R., Atkinson R., Brunekreef B., Cohen A., Forastiere F., Hurley F., Katsouyanni K., Krewski D., Krzyzanowski M. (2015). Quantifying the health impacts of ambient air pollutants: Recommendations of a WHO/Europe project. Int. J. Public Health.

[24] Hosseinpoor A.R., Forouzanfar M.H., Yunesian M., Asghari F., Naieni K.H., Farhood D. (2005). Air pollution and hospitalization due to angina pectoris in Tehran, Iran: A time-series study. Environ. Res..

[25] Erraguntla N.K., Sielken R.L., Valdez-Flores C., Grant R.L. (2012). An updated inhalation unit risk factor for arsenic and inorganic arsenic compounds based on a combined analysis of epidemiology studies. Regul. Toxicol. Pharmacol..

[26] Hänninen O., Knol A.B., Jantunen M., Lim T.A., Conrad A., Rappolder M., Carrer P., Fanetti A.C., Kim R., Buekers J. (2014). Environmental burden of disease in Europe: Assessing nine risk factors in six countries. Environ. Health Perspect..

[27] Hurley F., Hunt A., Cowie H., Holland M., Miller B., Pye S., Watkiss P. (2005). Methodology Paper (Volume 2) for Service Contract for Carrying Out Cost-Benefit Analysis of Air Quality Related Issues, in Particular in the Clean Air for Europe (CAFE) Programme.

[28] Abbey D., Petersen F., Mills P., Beeson W. (1993). Long-term ambient concentrations of total suspended particulates, ozone and sulfur dioxide and respiratory symptoms in a non-smoking population. Arch. Environ. Health.

[29] Ward D.J., Ayres J.G. (2004). Particulate air pollution and panel studies in children: A systematic review. Occup. Environ. Med..

[30] Beelen R., Raaschou-Nielsen O., Stafoggia M., Andersen Z.J., Weinmayr G., Hoffmann B., Wolf K., Samoli E., Fischer P., Nieuwenhuijsen M. (2013). Effects of long-term exposure to air pollution on natural-cause mortality: An analysis of 22 European cohorts within the multicentre ESCAPE project. Lancet.

[31] Nafstad P., Haheim L.L., Oftedal B., Gram F., Holme I., Hjermann I., Leren P. (2003). Lung cancer and air pollution: A 27 year follow up of 16,209 Norwegian men. Thorax.

[32] World Health Organization (2000). Air Quality Guidelines for Europe.

[33] Bostrom C.E., Gerde P., Hanberg A., Jernstrom B., Johansson C., Kyrklund T., Rannug A., Tornqvist M., Victorin K., Westerholm R. (2002). Cancer risk assessment, indicators, and guidelines for polycyclic aromatic hydrocarbons in the ambient air. Environ. Health Perspect..

[34] Bickel P., Friedrich R. (2005). ExternE—Externalities of Energy—Methodology.

[35] United States Environmental Protection Agency (US EPA) (2006). Integrated Risk Information System (IRIS). http://www.epa.gov/iris/.

[36] Takenaka S., Oldiges H., Konig H., Hochrainer D., Oberdorster G. (1983). Carcinogenicity of cadmium chloride aerosols in W rats. J. Natl. Cancer Inst..

[37] Peto J., Cuckle H., Doll R., Hermon C., Morgan L. (1984). Respiratory cancer mortality of Welsh nickel refinery workers. IARC Sci. Publ..

[38] Chovil A., Sutherland R., Halliday M. (1981). Respiratory cancer in a cohort of nickel sinter plant workers. Br. J. Ind. Med..

[39] Fewtrell L., Kaufmann R., Pruss-Ustun A. (2003). Lead Assessing the Environmental Burden of Disease at National and Local Levels.

[40] Schwartz J. (1994). Low-level lead exposure and children’s IQ: A meta-analysis and search for a threshold. Environ. Res..

[41] Pruss-Ustun A., Fewtrell L., Landrigan P.J., Ayuso-Mateos J. (2004). Lead exposure. Comparative Quantification of Health Risks. Global and Regional Burden of Disease Attributable to Selected Major Risk Factors.

[42] Hofstetter P. (1998). Perspectives in Life Cycle Impact Assessment: A Structured Approach to Combine Models of the Technosphere, Ecosphere, and Valuesphere.

[43] World Health Organization (WHO) (2004). Global Burden of Disease 2004 Update: Disability Weights for Diseases and Conditions.

[44] GBD (2016). Global Burden of Disease Study 2013 Results by Location, Cause, and Risk Factor.

[45] WIOŚ (2012). Air Quality Assessment in Mazovian Voivodship in the Year 2012.

[46] Tainio M., Juda-Rezler K., Reizer M., Warchałowski A., Trapp W., Skotak K. (2013). Future climate and adverse health effects caused by fine particulate matter air pollution: Case study for Poland. Reg. Environ. Chang..

[47] Adamkiewicz Ł., Badyda A.J., Gayer A., Mucha D. (2015). Disability-Adjusted Life Years in the Assessment of Health Effects of Traffic-Related Air Pollution. Adv. Exp. Med. Biol..

[48] Burnett R.T., Pope C.A., Ezzati M., Olives C., Lim S.S., Mehta S., Shin H.H., Singh G., Hubbell B., Brauer M. (2014). An Integrated Risk Function for Estimating the Global Burden of Disease Attributable to Ambient Fine Particulate Matter Exposure. Environ. Health Perspect..

[49] Lehtomäki H., Asikainen A., Rumrich I., Hänninen O. (2015). Ilmansaasteiden Tautitaakka Suomessa. ISTE-Raportti.

[50] Lanphear B.P., Hornung R., Khoury J., Yolton K., Baghurst P., Bellinger D.C., Canfield R.L., Dietrich K.N., Bornschein R., Greene T. (2005). Low-level environmental lead exposure and children’s intellectual function: An international pooled analysis. Environ Health Perspect..

[51] Asikainen A., Carrer P., Kephalopoulos S., de Oliveira Fernandes E., Wargocki P., Hänninen O. (2016). Reducing burden of disease from residential indoor air exposures in Europe (HEALTHVENT project). Environ. Health.

