# Supplementary material for: Burden of Mortality and Disease Attributable to Multiple Air Pollutants in Warsaw, Poland
Source: Int J Environ Res Public Health. 2017 Nov 8;14(11):1359. doi: 10.3390/ijerph14111359 (PMC5707998; doi:10.3390/ijerph14111359)
Supplement: Supplementary file 1 [file ijerph-14-01359-s001.pdf]

Burden of Mortality and Disease Attributable to Multiple Air Pollutants in Warsaw, Poland

Table S1. Primary and secondary pollutants considered

Primary pollutants	Secondary pollutants
SO_2 – sulfur dioxide	SO_4^{2-} – sulfate aerosol
NO_X – nitrogen oxides	NO_3^- – nitrate aerosol
PPM10 – primary PM, $\Phi \leq 10$ μm	
$PPM10\_{R}$ – re-suspended PPM10	
$PPM_{2.5}$ – primary PM, $\Phi \leq 2.5$ μm	
$PPM_{2.5\_R}$ – re-suspended PPM2.5	
CO – carbon monoxide	
C_6H_6 – benzene	
Pb – lead	
As – arsenic	
Cd – cadmium	
Ni – nickel	
BaP – benzo(a)pyrene	
**Particulate matter**	
$PM_{10} = PPM_{10} + PPM_{10\_R} + SO_4^{2-} + NO_3^-$	
$PM_{2.5} = PPM_{2.5} + PPM_{2.5\_R} + SO_4^{2-} + NO_3^-$	

Table S2. Population of Warsaw and Poland by age and sex. Population data for Poland was calculated from Global Burden of Disease 2013 country data for Poland, and population for Warsaw was based on the EEA (2016) and Central Statistical Office of Poland.

	Warsaw			Poland		
Age group	Males	Females	Males and Females	Males	Females	Males and Females
Under 5 years	49,564	46,771	96,335	1,038,348	980,613	2,018,961
5-9 years	39,347	37,521	76,868	858,840	810,173	1,669,013
10-14 years	36,900	35,199	72,098	906,055	856,835	1,762,890
15-19 years	42,936	41,488	84,424	1,146,896	1,098,475	2,245,371
20-24 years	55,704	55,284	110,987	1,316,457	1,261,049	2,577,507
25-29 years	72,428	75,220	147,648	1,768,330	1,717,835	3,486,165
30-34 years	75,336	79,689	155,025	1,647,587	1,606,209	3,253,795
35-39 years	65,739	68,741	134,480	1,486,777	1,455,461	2,942,238
40-44 years	49,911	51,882	101,793	1,129,578	1,111,889	2,241,467
45-49 years	46,691	51,168	97,858	1,080,145	1,085,429	2,165,573
50-54 years	57,617	67,431	125,048	1,440,683	1,506,341	2,947,024
55-59 years	62,915	76,582	139,497	1,432,517	1,591,721	3,024,238
60-64 years	50,245	64,015	114,261	1,378,466	1,607,523	2,985,988
65-69 years	28,989	40,471	69,461	546,339	709,920	1,256,259
70-74 years	24,608	38,989	63,597	543,561	815,406	1,358,967
75-79 years	20,892	35,949	56,841	426,206	719,273	1,145,479
80+ years	22,167	47,129	69,296	459,182	1,045,488	1,504,671
All age groups	801,988	913,529	1,715,517	18,605,966	19,979,639	38,585,605

Details on burden of disease calculation

Table S3. Disability weights and duration data.

Disease	Disability weight	Reference	Average duration (years)	Reference
Chronic bronchitis<br>(new cases)	0.05	Based on Hofstetter (1998).	40	Based on Hofstetter (1998).
Restricted activity days (RAD)	0.099	Lower respiratory infections (chronic sequelae) (World Health Organization, n.d.).	0.00274	Based on EboDe (Hänninen and Knol 2011).
LRS symptoms days (school children)	0.099	Lower respiratory infections, chronic sequelae (World Health Organization, n.d.).	0.00274	One day. Based on EboDe (Hänninen and Knol 2011).
LRS symptom days (adult)	0.279 (0.279-0.280)	Lower respiratory infections, chronic sequelae. (World Health Organization, n.d.).	0.00274	One day. Based on EboDe (Hänninen and Knol 2011).
Mild Mental Retardation (MMR) for children	0.36	EboDE (Hänninen and Knol 2011).	77.6	EboDE (Hänninen and Knol 2011).

Table S4. Summary of exposure-response functions (ERFs) used in the study. For details see text below.

Pollutant	Health endpoint	Age group	Type of ERF	ERF	References
PM2.5	Natural-cause mortality	30+	RR (relative risk)	1.062 (95% CI 1.040-1.083)	Héroux et al. 2015.
PM2.5, PM2.5-10	New cases of chronic bronchitis	30+	UR (unit risk)	5.33 x 10-5 (-0.17 x 10-5-11.3 x 10-5)	Hurley et al., 2005.
PM2.5	Restricted activity days (RADs)	15-64	UR	0.09 (0.079-1.013)	Hurley et al., 2005.
PM2.5, PM2.5-10	LRS days for school children	May-14	UR	0.186 (0.186-0.277)	Hurley et al., 2005.
PM2.5, PM2.5-10	LRS days for adults	15+	UR	0.13 (0.015-0.243)	Hurley et al., 2005.
NOx	Natural-cause mortality	30+	HR (hazard rate)	1.02 (95% CI 1.00-1.04)	Beelen et al. 2013.
SO2	Lung cancer	All	RR	1.01 (0.94-1.08)	Nafstad et al., 2003.
BaP	Lung Cancer	All	UR	8.7 x 10-5 (1.0 x 10-5– 10 x 10-5)	World Health Organization, 2000; Bostrom et al., 2002
Cd	Lung Cancer	All	UR	1.8 x 10-3 (1.0 x 10-3– 1.8 x 10-3)	Bickel and Friedrich 2005; Takenaka et al., 1983.
Ni	Lung Cancer	All	UR	2.4 x 10-4 (1.1 x 10-5 – 2.4 x 10-4)	Bickel and Friedrich 2005; Peto et al., 1984; Chovil et al., 1981.
As	Lung Cancer	All	All	0.00015	Erraguntla et al. 2012.
Pb	Mild Mental Retardation (MMR)	0-1	Specific, see text below for details.	Specific, see text below for details.	Fewtrell et al., 2003. See text below for details.
Pb	Cardiovascular disease	15-79	RR	Specific, see below for details.	Fewtrell et al., 2003. See text below for details.
CO	Ischemic heart disease	All	RR	1.00934 (1.01512-1.00359)	Hosseinpoor et al. 2005.
C6H6	Leukemia	All	UR	6.0 x 10-6 (2.26 x 10-6 - 7.8 x 10-6)	Hänninen and Knol 2011.

Table S5. Background burden in the study area**.**

Disease	Age group	Burden measure	#	Factor	Details
All causes	All	DALY	536,529	-	Total (All causes).
All causes	All	Deaths	18,254	-	Total (All causes).
Non-accidental mortality	30+	YLL	267,244	PM2.5	Communicable, maternal, neonatal, and nutritional disorders; Non-communicable diseases
				NOx	
Non-accidental mortality	30+	Deaths	17,016	PM2.5	Communicable, maternal, neonatal, and nutritional disorders; Non-communicable diseases
				NOx	
Lung cancer	All	DALY	23,373	SO2	Trachea, bronchus, and lung cancer.
Lung cancer	All	Deaths	1,081	SO2	Trachea, bronchus, and lung cancer.
Ischemic heart disease	All	DALY	52,377	CO	Ischemic heart disease.
Ischemic heart disease	All	Deaths	3,487	CO	Ischemic heart disease.
Ischemic heart disease	15-79	DALY	40,456	Pb	Ischemic heart disease.
Ischemic heart disease	15-79	Deaths	1,759	Pb	Ischemic heart disease.
Cerebrovascular disease	15-79	DALY	27,732	Pb	Cerebrovascular disease.
Cerebrovascular disease	15-79	Deaths	1,350	Pb	Cerebrovascular disease.
Hypertensive heart disease	15-79	DALY	4,427	Pb	Hypertensive heart disease.
Hypertensive heart disease	15-79	Deaths	188	Pb	Hypertensive heart disease.
Other cardiac diseases	15-79	DALY	13,097	Pb	*
Other cardiac diseases	15-79	Deaths	481	Pb	*

*) Cardiomyopathy and myocarditis; Atrial fibrillation and flutter; Aortic aneurysm, Peripheral vascular disease; Endocarditis, Other cardiovascular and circulatory diseases.

Particulate matter (PM2.5, PM2.5-10)

Natural-cause mortality. The Years of Life Lost (YLLs) due to PM2.5 were estimated with the following equation:

$$PAF= (RR^{(1/Eb)})^{E} \quad (S1)$$

$$YLL = PAF \times YLL_{\text{Natural-cause mortality}} \quad (S2)$$

Where PAF is the population attributable fraction, RR is the relative risk for natural-cause mortality per 10 μg/m3 change in PM2.5 exposure, E is the exposure for PM2.5 (unit μg/m3), Eb is the PM2.5 exposure increment to which the RR is related (10 μg/m3), $YLL_{\text{Natural-couse mortality}}$ is the background YLLs due to natural-cause mortality, and the YLL is the disease burden caused by PM2.5 related mortality.

For the RR a value of 1.062 (95% confidence interval (CI) 1.040-1.083) was used, based on the recommendation of the World Health Organization (WHO) project ''Health risks of air pollution in Europe—HRAPIE'' (Héroux et al. 2015).

The background YLL data included YLLs caused by the communicable, maternal, neonatal, and nutritional disorders, and non-communicable diseases (Table S4, Supplementary material). In Héroux et al. (2015) the health outcomes for this recommended RR were defined to be all-cause mortality (natural) for age 30+. Therefore we excluded injuries and other accidents from the background YLLs and we assumed that PM2.5 will increase mortality for the age group 30, or older.

New cases of chronic bronchitis, RADs, LRS symptoms days for school children and LRS symptoms days for adults. The Years Lost due to Disabilities (YLDs) for morbidity outcomes were estimated with equations:

$$AI_k = E_k \times UR_k \quad (S3)$$

$$DALYk=AI_k \times DW_k \times D_k \quad (S4)$$

Where UR_k is the unit risk for disease k, AI_k is the Attributable Incidence (number of new cases per year) for disease k, E is the exposure level (PM2.5 or PM2.5-10), DW_k is the Disability Weight for disease k, and D_k is the duration of condition in years for disease k.

The unit risk values for different morbidity outcomes were adopted from the Clean Air for Europe (CAFE) program report (Hurley et al., 2005). The URs are summarized in Table S3 (Supplementary material) and the DWs and Ds in the Table S2 (Supplementary material). The UR uncertainty was quantified with triangular distributions and based on the values from the CAFE report. For the LRS days for adults, 30% of the adult population was estimated to have chronic respiratory symptoms, with uncertainty range from 20% to 50% (Hurley et al., 2005).

Nitrogen oxides (NOx)

The YLLs due to exposure to NOx was estimated with equations S1 and S2 by using the HR value of 1.02 (95% CI 1.00-1.04) per 20 μg/m3 change in NOx concentration. The HR was based on Beelen et al. (2013) cohort study and the YLL impact was calculated using equations S1 and S2.

Sulfur dioxide (SO_2)

The DALYs due to SO2 related lung cancers were estimated with following equations:

$$RR = \exp(E \times \ln(RR)/Eb) \quad (S5)$$

$$PAF = (RR - 1) / RR \quad (S6)$$

$$DALY = PAF \times DALY_{Lung\ cancer} \quad (S7)$$

Where RR is relative risk per 10 μg/m3 changes in SO2 exposure, E is the exposure for SO2, Eb is the exposure increment to which the RR is related (10 μg/m3), PAF is population attributable fraction and $DALY_{Lung\ cancer}$ is the background lung cancer DALY in the study area. The RR was adopted from Nafstad et al. (2003) study that followed a cohort of 16 209 Norwegian men with 27 year follow up time. The resulting RR associated to 10 μg/m3 change in SO2 concentration was 1.01 (95% CI 0.94-1.08) (Nafstad et al., 2003).

Benzo(a)pyrene (BaP)

The DALYs due to lung cancer caused by BaP was calculated with following equations:

$$AI = E \times UR \times Pop \quad (S8)$$

$$DALY = (AI/75)*DALY_b/death_b \quad (S9)$$

Where E is exposure to BaP (unit ng/m3), UR is a life time cancer risk of lung cancer, Pop is the size of the study population and AI is the Attributable Incidence (number of new cases per year). The life time cancer risk of the population was divided with the 75 years to estimate new cases of cancers per year by assuming the average life span of 75 years. The number of new cancer cases per year was converted to DALYs by multiplying the number of cases with the mean DALY loss of one lung cancer (21.6 DALYs per lung cancer death), estimated from the GBD 2013 data for Poland (Global Burden of Disease Study 2013).

For the UR a value of 8.7 x 10-5 cancers per ng/m3 exposure to BaP was used, based on the WHO Air Quality guidelines for Europe (World Health Organization, 2000). The upper and lower bound values of 10 x 10-5 per ng/m3 and 1.0 x 10-5 per ng/m3, respectively, were used based on the summary of risk estimates for BaP from Bostrom et al. (2002) review.

Cadmium (Cd)

The DALYs due to lung cancer caused by lifetime exposure to Cd were estimated with UR approach by adopting the equations S8 and S9. For UR a value of 1.8 x 10-3 cancers per μg/m3 was used. The same unit risk value was used in the ExternE year 2005 update (Bickel and Friedrich, 2005). For upper and lower band values of 9.2 x 10-2 and 1.0 x 10-3 were used, based on the Takenaka et al. (1983) and author judgment, respectively.

Nickel (Ni)

The DALYs due to lung cancer cases caused by lifetime exposure to Ni were estimated with UR approach by adopting the equations S8 and S9. For UR a value of 2.4 x 10-4 cancers per μg/m3 was used. The same UR was used in ExternE (Bickel and Friedrich, 2005) and it is based on the inhalation UR value from United States Environmental Protection Agency (US EPA) Integrated Risk Information System (IRIS) database (United States Environmental Protection Agency, n.d.). For upper and lower bounds values of 4.6 x 10-4 and 1.1 x 10-5 were used, based on Peto et al. (1984) and Chovil et al. (1981), respectively.

Arsenic (AS)

The DALYs due to lung cancer cases caused by lifetime exposure to As were estimated with UR approach by adopting the equations S8 and S9. For UR a value of 0.00015 cancers per μg/m3 was used based on a combined analysis of three epidemiological studies (Erraguntla et al. 2012).

Lead (Pb)

The adverse health effects of Pb exposure were estimated by following the WHO burden of disease guidelines for lead (Fewtrell et al., 2003). Due to the non-linear nature of the dose-response relationship between Pb and the associated health effects, the total health burden due to Pb in the study area was first calculated and then the fraction of that burden due to air pollution related Pb was estimated.

The average Pb concentrations in the air were converted to blood Pb levels by assuming that 1.0 μg/m3 increase of Pb in the air leads to 50 μg/L increase in blood level Pb levels, following a similar approach taken in the ExternE (Bickel and Friedrich, 2005). For the sensitivity analysis +/-25% uncertainty around this conversation factor was estimated. The background blood Pb levels for children and adults were estimated based on the local exposure study and to WHO recommended values, respectively (see below for details).

Mild Mental Retardation (MMR). The exposure to Pb in early childhood has been associated with the decreased intelligence (Lanphear et al., 2005). For some individuals the decrease in the intelligence quotient (IQ) due to Pb leads to MMR. In (Fewtrell et al., 2003) mental retardation was estimated to be mild when IQ drops below 70 points.

Table S6. Calculation of Mild Mental Retardation (MMR) due to Pb. The calculations follows the methods presented in the (Fewtrell et al., 2003).

Blood lead level intervals	Proportion of children at risk (H)	Fraction of population in IQ interval (I)	Fraction of population at risk with exposure (H x I)	Adjustment factor for EUR-B area	Number of 0-1 year old children in Warsaw,	Number of new cases of MMRs per year (J x K x L)
<5 µg/dl	0.737	-	-	-	-	-
5-10 µg/dl	0.253	0.24	0.00061	-	-	-
10-15 µg/dl	0.01	0.8	0.00008	-	-	-
15-20 µg/dl	0	1.45	0	-	-	-
20> µg/dl	0	1.59	0	-	-	-
**Total**	**-**		**0.00069**	**1.53**	**18566**	**19.52**

The background blood level Pb concentrations in children were estimated from the Barton (2011). In that study the Pb and Cd levels in blood, hair and teeth were measured for 300 preschool age children in Southern Poland. The geometric mean blood level Pb concentrations were 42 µg/l and geometric standard deviation 1.5 for children living in urban area (n=99). This is close to the regional blood level of 58 µg/l for children in Poland, Turkey and Yugoslavia, presented in the appendix of the (Fewtrell et al., 2003).

By assuming the blood level geometric mean concentration of 42 µg/l and variation with the geometric standard deviation of 1.5, the children were divided into five different exposure groups based on the lead levels in their blood (Table S5, Supplementary material). The proportion of children at risk in each exposure group was then multiplied with the fraction of population in each IQ interval (based on Table 2 in Fewtrell et al. (2003) and the review by Schwartz (1994)). The IQ intervals represent the fraction of population that could potentially develop MMR if their IQ would decrease as a result of Pb exposure. For example, with the exposure group of 5-10 µg/dl, the background IQ interval in risk is in between 70.00 and 70.65 IQ points, and these children would develop MMR if their IQ would drop due to Pb more than 0.65 IQ points.

The fraction of the population at risk of developing MMR in the study area was calculated by first summing at risk population in each exposure intervals and then multiplying the fraction with the regional adjustment factors. Regional adjustment factors are used to take into account that several other stressors are causing MMR and by using the adjustment factor the combined effect of Pb and these other stressors can be estimated. For Poland the adjustment factor is 1.53 (EurB-group in Table 3, (Fewtrell et al., 2003)). The number of new

MMR cases per year is then calculated by multiplying the adjusted at-risk population with the number of 0-1 year old children in the study area. To calculate the DALYs, the number of new MMR cases per year was multiplied with the disability weight of 0.36 and with the average duration of 77.6 years (Table S2, Supplementary material).

By converting the annual average Pb concentration in the air to blood level concentration by using the conversation factor from ExternE (Bickel and Friedrich, 2005) study (see details earlier), 2.4% of total burden of Pb for children was estimated to be due to air pollution emissions from local transport.

Cardiovascular disease. The calculation of new cases of cardiovascular diseases due to Pb followed similar pattern as the calculation of MMRs for children. For the background blood level concentrations, the regional blood level of 9.2 μg/dl was used with the standard deviation of 3 for adults in Poland, Turkey and Yugoslavia, as presented in the appendix of the Fewtrell et al. (2003). Based on this background exposure, population was divided into five exposure groups using the same method as was used in the MMR calculations (Table S5, Supplementary material).

Table S7. Relative risk values for cardiovascular disease for different blood level Pb's. Adopted from (Fewtrell et al., 2003) and based on (Pruss-Ustun et al., 2004).

**Gender, disease**	**Age group**				
**Male, <5 μg/dl**	**15–29**	**30–44**	**45–59**	**60–69**	**70–79**
Ischaemic heart disease	1.000	1.000	1.000	1.000	1.000
Cerebrovascular disease	1.000	1.000	1.000	1.000	1.000
Hypertensive disease	1.000	1.000	1.000	1.000	1.000
Other cardiac diseases	1.000	1.000	1.000	1.000	1.000
**Male, 5-10 μg/dl**					
Ischaemic heart disease	1.041	1.041	1.032	1.018	1.014
Cerebrovascular disease	1.056	1.056	1.044	1.029	1.020
Hypertensive disease	1.122	1.122	1.059	1.036	1.027
Other cardiac diseases	1.013	1.013	1.009	1.006	1.003
**Male, 10-15 μg/dl**					
Ischaemic heart disease	1.130	1.130	1.100	1.055	1.043
Cerebrovascular disease	1.177	1.177	1.137	1.089	1.061
Hypertensive disease	1.413	1.413	1.189	1.111	1.083
Other cardiac diseases	1.039	1.039	1.026	1.017	1.010
**Male, 15-20 μg/dl**					
Ischaemic heart disease	1.225	1.225	1.172	1.093	1.072
Cerebrovascular disease	1.312	1.312	1.239	1.152	1.104
Hypertensive disease	1.779	1.779	1.334	1.192	1.142
Other cardiac diseases	1.067	1.067	1.044	1.029	1.017
**Male, 20> μg/dl**					
Ischaemic heart disease	1.276	1.276	1.210	1.112	1.087
Cerebrovascular disease	1.385	1.385	1.293	1.185	1.126
Hypertensive disease	1.996	1.996	1.413	1.235	1.172

Other cardiac diseases	1.081	1.081	1.053	1.035	1.02
**Female <5 μg/dl**	**15–29**	**30–44**	**45–59**	**60–69**	**70–79**
Ischaemic heart disease	1.000	1.000	1.000	1.000	1.000
Cerebrovascular disease	1.000	1.000	1.000	1.000	1.000
Hypertensive disease	1.000	1.000	1.000	1.000	1.000
Other cardiac diseases	1.000	1.000	1.000	1.000	1.000
**Female, 5-10 μg/dl**					
Ischaemic heart disease	1.026	1.026	1.021	1.011	1.009
Cerebrovascular disease	1.035	1.035	1.028	1.018	1.013
Hypertensive disease	1.076	1.076	1.038	1.023	1.017
Other cardiac diseases	1.008	1.008	1.005	1.004	1.002
**Female, 10-15 μg/dl**					
Ischaemic heart disease	1.081	1.081	1.063	1.035	1.027
Cerebrovascular disease	1.11	1.11	1.086	1.056	1.039
Hypertensive disease	1.247	1.247	1.117	1.07	1.052
Other cardiac diseases	1.025	1.025	1.017	1.011	1.006
**Female, 15-20 μg/dl**					
Ischaemic heart disease	1.139	1.139	1.107	1.058	1.046
Cerebrovascular disease	1.19	1.19	1.147	1.095	1.065
Hypertensive disease	1.446	1.446	1.203	1.119	1.088
Other cardiac diseases	1.042	1.042	1.028	1.018	1.011
**Female, 20> μg/dl**					
Ischaemic heart disease	1.169	1.169	1.13	1.07	1.055
Cerebrovascular disease	1.232	1.232	1.179	1.115	1.079
Hypertensive disease	1.556	1.556	1.248	1.145	1.107
Other cardiac diseases	1.051	1.051	1.033	1.022	1.013

Increased systolic blood pressure is associated with an increase in four different diseases: ischemic heart disease, cerebrovascular disease, hypertensive disease and other cardiac diseases. The RRs for each disease, exposure interval, age group and gender were obtained from Fewtrell et al. (2003) (Table S6, Supplementary material). By combining the exposure data with the RRs DALYs were calculated with the following equations:

$$PAF_{j,k} = (\sum_l(P_l x RR_{l,j,k})-1)/ \sum_l(P_l RR_l) \qquad (S10)$$

$$DALY = DALY_k \ x \sum j(PAF_{j,k}) \qquad (S11)$$

Where P_l is the fraction of population in at exposure interval l, $RR_{l,j,k}$ is the relative risk at exposure interval l, gender j and disease k, and $DALY_k$ is the background DALY for disease k. By converting the annual average Pb concentration in the air to blood level concentration by using the conversation factor from the ExternE (Bickel and Friedrich, 2005) study (see details earlier), 10.2% of the total burden of Pb for adults was estimated to be due to air pollution emissions from local transport.

Carbon monoxide (CO)

The change in ischemic heart disease due to chronic exposure to CO was estimated based on the RR from the Hosseinpoor et al. (2005) study that estimated RR between CO and angina pectoris admissions in Tehran, Iran. The resulting RR was 1.00934 with 95% CI from 1.00359 to 1.01512 per 1000 μg/m3 change in CO concentration. The calculation was done using equations S1 an S2.

Benzene (C6H6)

For C6H6 we adopted the unit risk value for leukemia from Hänninen and Knol (2011) study that estimate the burden of disease due to environmental stressors in Europe. The mean UR was 6 × 10-6 cases of leukaemia per 1 μg m-3 in life time and the uncertainty from 2.2 x 10-6 to 7.8 x 10-6 per 1 μg m-3. The DALYs were calculated by assuming that one death due to leukaemia will cause 22.3 DALYs, based on the Global Burden of Disease 2013 data for Poland.

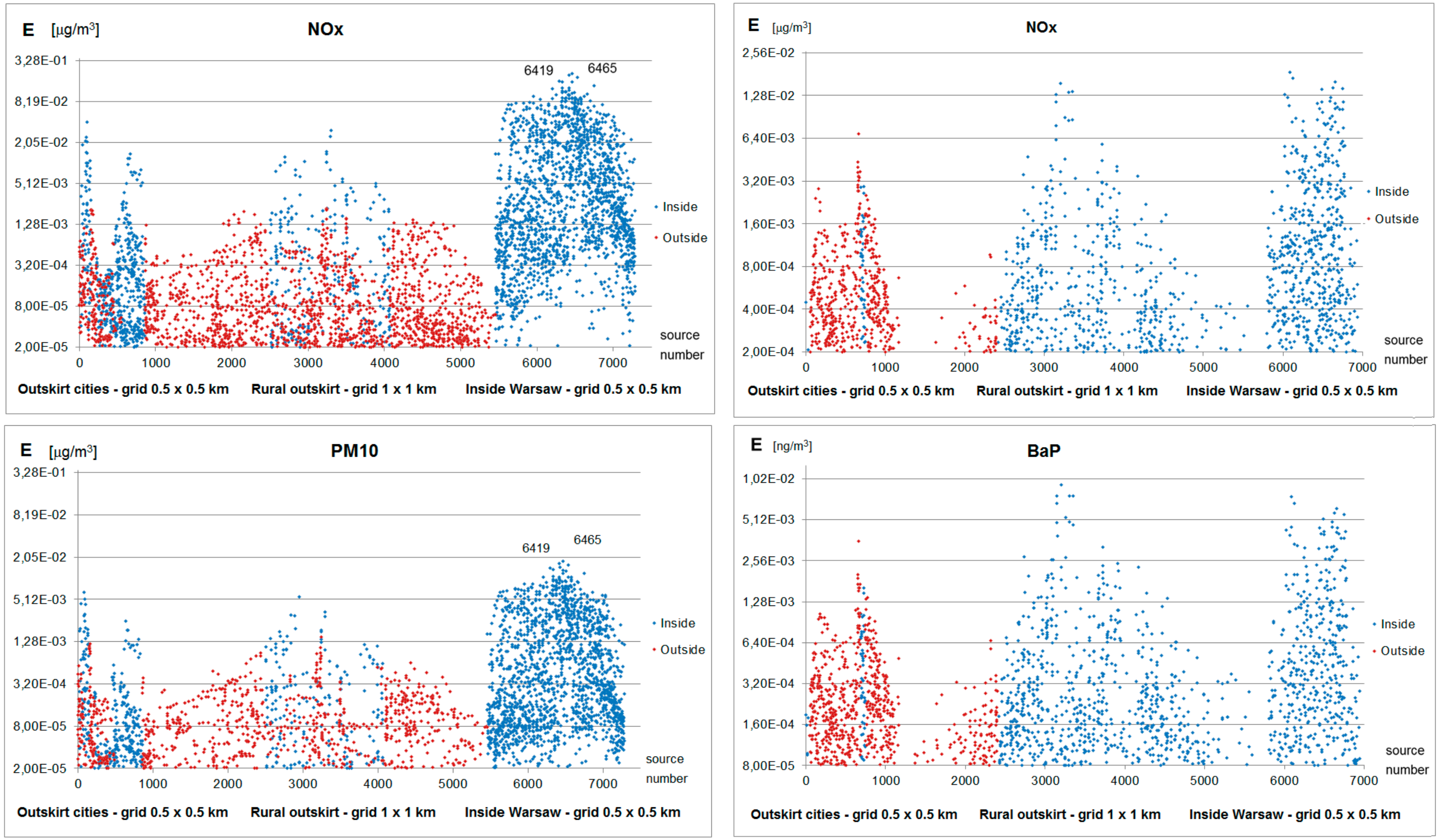

Figure S1. Exposure of the selected pollutants attributed to the individual emission sources. X-axis – the number of the source, Y-axis – the exposure in logarithmic scale. Left panels – 7285 line sources, Right panels – 6962 area sources. Positions of the sources and the related grid resolution are explained below the X-axis description.
